# A Simple and Effectual Plan for the Prevention of the Malignant Cholera

**Published:** 1832-10

**Authors:** 


### BIBLIOGRAPHICAL NOTICES.

*A Simple and Effectual Plan for the Prevention of the Malignant Cholera.* By JOHN BURNE, M.D., Physician to the Public Dispensary, Chancery Lane.—8vo. pp. 4.

*Observations on Spasmodic Cholera, its Origin, Nature, and Treatment; with Remarks on Epidemic Diseases generally.* By HENRY M'CORMAC, M.D., Member of the Royal College of Surgeons, Edinburgh; Physician to the Belfast Fever Hospital, and Physician to the Cholera Hospital. Second Edition.—8vo. pp. 28. Longman and Co.

*A Letter addressed to the Central Board of Health; written with the View of establishing Rational Principles for the Treatment of Cholera, and shewing the Danger of the Mode of Practice at present generally followed.* By JOHN GEORGE FRENCH, Surgeon to the St. James's Cholera Hospital, &c.—8vo. pp. 22. Rivington's.

*A Treatise on Cholera; containing the Author's Experience of the Epidemic known by that name, as it prevailed in the City of Moscow, in Autumn* 1830 *and Winter* 1831. By JAMES KEIR, M.D. &c. &c.—8vo. pp. 138. Black, Edinburgh; and Longman, London.

*Experimental Inquiries in Chemical Pathology. Part I. (complete in one volume,) on the Blood: with an Appendix, containing Remarks on the Nature and Treatment of Cholera Asphyxia.* By HORATIO PRATER.—8vo. pp. 304. Highley, London.

OH, *cholera! cholera!* quæ te dementia cepit? No sooner have we one mass of cholera pamphlets placed in our critical alembic, in order to extract their essence for presentation to our readers, than another overwhelms our table; so that we are sometimes almost tempted to make one serve to heat the furnace for the distillation of another: and, were we to do so, many of them would no doubt be saved from a more dishonourable destiny. Few, very few, (not even excepting the puny pamphlet of Dr. BURNE,) seem made to sell, and still fewer can have been seriously made to read; or else we should not find our old friends Etymology and Syntax so utterly despised. In one place we find an author modestly begins his preface in the third person; but, at the end of the second sentence, his egotism can no longer be restrained, and the modest *he* is changed to the boasting *I*; and in another, to display his classical attainments, he confounds the mental disorder of the Homoopath Hahneman with a corporeal disease of the scapula, writing *omo-* for *homo-*opathic. But let this, and much more like it, pass, for the less that is said about such things the better; and in mercy we turn to those in which some really useful matter is contained, and with which we head our pages.

Dr. M'Cormac's is a valuable and well-written pamphlet, in which the question of contagion is calmly discussed, and in which he endeavours to shew that cholera, and other epidemic diseases, are general benefits, even if they inflict some partial hardships on particular individuals: he says,

"If we look around us and reflect, we observe that the most extraordinary care has been taken by nature to secure the health of our species. Every encouragement is held out to that line of conduct by which it is promoted, while misery and unhappiness are the portion of him who neglects it. Nature protects the healthy, while she cuts off the diseased, and thus averts the degradation of our race; but her ordinary means of doing this are not always sufficient: some circumstance is sure to lead to the production of a pestilence, which stretches forth its ruthless hand till the law of its existence is accomplished. The healthy and the vigorous do sometimes, it is true, fall victims; but nothing can be better ascertained than that the debilitated and the unsound do, for the most part, perish alone. If mankind were healthy and vigorous, pestilence would never assail them; it could not do so. It is commonly and truly said, that the pestilence knows its victim; it can have no mission to the healthy. What a lesson to mankind, then, if they will but take it; what an incitement to temperance and sobriety, to procuring the means of health and well-being to ourselves and to others. We may mourn over the melancholy fate of humanity, subject to such a complication of evils; but it is, on the whole, well. The population of the earth is thus kept young and vigorous, and any evils which we suffer from such a condition of things here will, I trust, be amply overbalanced hereafter." (P. 4.)

With regard to the communicability of the disease from man to man, we have already given a condensed account of the most important arguments on both sides of the question, and therefore there is the less occasion for discussing the problem now; still we cannot refrain from making a short extract from Dr. M'Cormac's pages, as he appears to have had ample opportunities of observing the disease, and to have come unprejudiced to the inquiry.

"No subject has so frequently and so anxiously been discussed, as whether epidemic diseases in general, and cholera in particular, be contagious. Men of the highest talents and most extensive experience, both professional and unprofessional, have taken opposite views. A multitude of indirect arguments have been urged on both sides, with more or less weight, and with more or less skill, according to the talents of the disputants. The direct arguments may be reduced, however, to two: the contagionists affirming that many persons have taken the disease after coming into contact with it, and the anti-contagionists asserting that many are exposed to the disease who do not take it at all. Now, both are correct; but if the former can be ascertained, in a sufficient number of instances, to do away with the mere coincidence, then the contagious nature of cholera (for I speak of cholera,) is ascertained beyond a doubt; and I believe the evidence in favor of the disease having arisen from intercourse to be overwhelming. It would take up too much room to consider the collateral evidence in this question, in this place; nor is it necessary; for if it be once established that, in one or more instances, cholera has resulted from personal communication, then the question is settled beyond appeal. If the facts stated on this head be correct, there is no possibility of arriving at any other conclusion: and facts of this kind have been transmitted from a variety of quarters, and from individuals of scientific correctness and unimpeachable veracity.* Nor did I myself yield my conviction on the subject, till the number and the urgency of the instances which I witnessed seemed to leave no room for further hesitation." (P. 6.)

* "The earliest case of cholera in Belfast was that of a person who had arrived from Glasgow: the particulars, which I witnessed myself, are fully stated in the Report of the Board of Health. Instance after instance has occurred to my own knowledge, wherein persons contracted the disease after coming in contact with those who had it. In the numerous list of English and French publications which I have consulted, and to the authors of which I acknowledge my obligations in general terms, numerous cases of the transmission of the disease from person to person are given. These are too numerous to quote; but I refer the reader to Orton, Hawkins, Kennedy, Hancock, the Lancet, the Edinburgh Medical Journal, the Foreign Quarterly, the Westminster, and many others. Mr. Bell, and the conductors of many of our excellent periodicals, question the point of contagion. It is truly remarkable how such a difference of opinion can exist upon this subject. I have seen many who were attacked after washing the remains of their friends, or visiting their relatives in the hospitals. The woman who washed the remains of a gentleman in Belfast, (Mr. H., who died of cholera,) came into the hospital, in collapse, and died. A man came to Belfast from Dromore: while in Belfast, he helped a cholera patient into the vehicle used to convey the sick to the hospital: he then returned to Dromore, and died, within twenty-four hours, of cholera. His daughter, who attended him in his last moments, contracted cholera herself, but recovered. These were the first cases in Dromore; the girl had not left home. This was related to me by an eye-witness of the events, a clergyman in Dromore, whose word is perfect authority. The first case in Portaferry was that of a man who came sick from Liverpool, by the steam-boat: he died in Portaferry. After his death, a friend performed some of the last offices to his remains. This friend was servant-man to a gentleman in Portaferry: the gentleman took the disease, but recovered. A most respectable practitioner, who attended the gentleman, related this to me. In the Cholera Hospital of Belfast, all the servants have been ill, some of them for the second or third time. One nurse narrowly escaped dying, as she fell into collapse. The man who drove the cholera carriage fell into collapse, and died in a few hours: he had not reported his case early. One of the apothecaries had a smart attack, but recovered. I had the premonitory symptoms, with acute pains in my stomach, and faintishness, which I subdued by the timely use of medicine. In the hospital in Ballymacarett, a suburb of Belfast, the first physician appointed took cholera, and died: circumstances prevented me from seeing this unfortunate gentleman, who was of a delicate constitution, and, moreover, did not attend to his case early. He

Mr. French's Letter is a fit companion for Dr. M'Cormac's Essay; it is a short, pithy, and sensible production: we only regret that the writer did not detail some more cases in which the treatment he recommends was pursued; because, on inquiry, we find that he could have done so, as they have been numerous, and some very decided and important. His object, however, was rather to enunciate certain principles than to empirically recommend any particular practice; and this he has done in a manner as brief as possible. His opinion is,

"That the alimentary canal becomes subjected to a process which altogether supersedes digestion, and, by this process, a large quantity of fluid is produced as an excretion, which rapidly diminishes the bulk of the blood: with the mechanism of this production we are as yet unacquainted.

"That this constitutes the disease.

"That in some instances the disease continues its progress till the death of the patient; but in the very large majority of cases, when left to nature, the disease ceases when it has produced a state of collapse, varying in intensity.

"That this state of collapse is remarkable in the phenomenon that the patient himself complains of heat felt throughout his body, while to the bystander he appears to be perfectly cold.

"That extreme thirst attends this state of collapse.

"That, though fluids, requiring digestion, are rejected by the stomach, and therefore with difficulty find their way into the system, cold water, which is most grateful to the patient, becomes gradually received and retained.

"That the blood, in consequence of a change in its constituent principles, by which it becomes more viscid, is thereby rendered less easy of circulation.

"That, though the evacuations of the morbid excretion cease, retching continues, which assists, mechanically, the passage of the blood, and produces a general relaxation of the system; hence diminishing the obstacles to the circulation of this fluid.

"That when the system becomes replenished with fluid to a certain extent, and the circulation of the blood thus acquires a certain degree of energy, the gall-duct, which remained closed during the did not believe in contagion; and I was told that, when pressed with fatigue, he sometimes slept on beds which had been occupied by patients. Indeed, he had been known to yield his own bed to them. His successor also took ill, and recovered after the most energetic medication. These facts are known to all here, and they are only a part of those which have come to my knowledge. The season is calm and beautiful, and the air pure: how can we suppose that any possible variation now existing in it could, in this country, produce such an epidemic? If it were so, why are not people attacked in the country? why are the attacks not simultaneous? Paris, where the disease broke out so rapidly, is a consummation of filth; and the people have incessant intercourse in their theatres, *cafés*, gardens, *lieux publiques*, &c. I am of opinion that no case of cholera has occurred in Europe, and I may now add America, which was not the exclusive result of contagion.

progress of the disorder, now permits the flow of the bile. The bile, generally in a vitiated state, becomes ejected by vomiting, (this action still assisting the circulation;) a further flow of bile then gradually prepares the alimentary canal for its proper functions.

"That, though no specific fever follows the disease, the shock thus given to the system commonly produces the most severe effects, in the form of local inflammations and congestions in the various viscera." (P. 3.)

The important points here laid down are two; one practical, one physiological: viz. 1st, that the copious administration of cold water is the most efficient remedial means; and, 2dly, that the secretions are not wholly and essentially suspended. We are obliged to Mr. French for his letter, and, as we have said, only regret that he has not been a little more liberal of his words.

Dr. Keir's volume may be read with pleasure and advantage; but as it relates to points which have already, in so many reviews, been brought before our readers, we do not find much regarding the ancient history of the disease that demands an extract now; and even the ravages of cholera at Moscow shrink into comparative unimportance, since our attention is chiefly fixed (and very properly) upon its progress nearer home. One passage, however, as tending to confirm the statements made by Dr. M'Cormac respecting the communicability of the disease, we cannot forbear extracting: for, although admitting the probability of a peculiar state of the atmosphere, predisposing the constitution to become affected, and mentioning the difference of opinion that exists as to the contagious nature of cholera, he says,

"Through the course of the Volga, wherever the disease appeared, it seemed to begin with, or to be communicated by, the men employed in navigating the barks on the river: at Kfalinsk, a town on the Volga, two men were employed in mowing grass on a little island in the river, when one of them was seized with this disease. On examination, it was found that they had been in company with the people who navigate the barks. The disease then spread through the town. At Saratoff, the first man affected was one of these people, who was found lying ill with the complaint on the banks of the Volga; the police officer who examined him was soon thereafter himself affected. Drs. Kildonchefsky and Zabiakin, who were sent to Saratoff and Kfalinsk, agree in believing that the disease was communicated to the inhabitants of these towns by the people who navigate the barks of the Volga."

We might extract more to the same purpose, but it is unnecessary; for this is not a case in which the cumulative argument can do more than settle the degree, and this we had better search for in cases nearer home; in such as those which have been recorded by Dr. Velpeau, in the Archives de Medicine. This physician, our readers will recollect, was one of those who signed the sudden manifesto, that cholera should not be (for, at that early epoch, it was absurd to say that it had not been,) contagious in France; and finding the error into which he had been betrayed, he has now very honourably confessed it, and published an account of upwards of eighty cases, jealously examined, that afford the most conclusive proof of the contagious nature of the disease.

Mr. PRATER's Experimental Inquiries in Chemical Physiology seem to have been pursued with much ardour, and no pains spared: we cannot, however, but lament that the work is so far deficient in the "lucidus ordo" as to make it a toil to read it, and this notwithstanding the interesting nature of the inquiry to which it has been devoted.

Animal chemistry is still in such a rudimental state, that every contribution must be esteemed of value; and those which have lately been carried on by Dr. O'SHAUGHNESSY, our author, and others, to further our knowledge of the constitution and properties of the blood, although they have not as yet led to any very important improvements in the treatment of cholera, still possess at this moment much interest; especially as the saline injections were suggested by the facts these chemical researches have revealed. Much, however, here yet remains to be done, and part of this we recommend our author to attempt to do: the object is worthy any one's pursuit, and, even if he fail, the attempt itself is creditable.

---

